# Differences in dietary composition and preference maintained despite gene flow across a woodrat hybrid zone

**DOI:** 10.1002/ece3.7399

**Published:** 2021-03-17

**Authors:** Danny P. Nielsen, Marjorie D. Matocq

**Affiliations:** ^1^ Department of Natural Resources and Environmental Science University of Nevada Reno NV USA; ^2^ Graduate Program in EECB University of Nevada Reno NV USA

**Keywords:** adaptation, detoxification, herbivore, hybridization, *Neotoma*, toxin tolerance, woodrat

## Abstract

1. Ecotones, characterized by adjacent yet distinct biotic communities, provide natural laboratories in which to investigate how environmental selection influences the ecology and evolution of organisms. For wild herbivores, differential plant availability across sharp ecotones may be an important source of dietary‐based selection.
2. We studied small herbivore diet composition across a sharp ecotone where two species of woodrat, *Neotoma bryanti* and *N*. *lepida*, come into secondary contact with one another and hybridize. We quantified woodrat dietary preference through trn*L* metabarcoding of field‐collected fecal pellets and experimental choice trials. Despite gene flow, parental *N. bryanti* and *N. lepida* maintain distinct diets across this fine spatial scale, and across temporal scales that span both wet and dry conditions.
3. *Neotoma bryanti* maintained a more diverse diet, with *Frangula californica* (California coffeeberry) making up a large portion of its diet. *Neotoma lepida* maintains a less diverse diet, with *Prunus fasciculata* (desert almond) comprising more than half of its diet. Both *F. californica* and *P. fasciculata* are known to produce potentially toxic plant secondary compounds (PSCs), which should deter herbivory, yet these plants have relatively high nutritional value as measured by crude protein content.
4. *Neotoma bryanti* and *N. lepida* consumed *F. californica* and *P. fasciculata*, respectively, in greater abundance than these plants are available on the landscape—indicating dietary selection. Finally, experimental preference trials revealed that *N. bryanti* exhibited a preference for *F. californica*, while *N. lepida* exhibited a relatively stronger preference for *P. fasciculata*. We find that *N. bryanti* exhibit a generalist herbivore strategy relative to *N. lepida*, which exhibit a more specialized feeding strategy in this study system.
5. Our results suggest that woodrats respond to fine‐scale environmental differences in plant availability that may require different metabolic strategies in order to balance nutrient acquisition while minimizing exposure to potentially toxic PSCs.

## Graphical abstract

Hybridization can homogenize gene pools of species that come into secondary contact, yet species differentiation may persist despite gene flow. We studied dietary ecology of two closely related and hybridizing species of woodrat. We found that dietary differences are maintained across seasons and during both wet and dry periods.

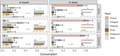

## 1 INTRODUCTION

Ecotones are characterized by spatial transition in environmental variables that can create selective gradients that generate or maintain diversity (Smith et al., 1997). When sharp abiotic gradients support the establishment of spatially proximate but distinct vegetation communities (Walker et al., 2003), animals must respond to abrupt spatial transitions in abiotic and biotic resources. Such spatially proximate, yet dissimilar selective environments have the potential to generate or reveal the ecological adaptations or forms of phenotypic plasticity that permit species to exist in disparate environments (Ghalambor et al., 2007; West‐Eberhard, 1989).

At sharp environmental transitions, one of the primary challenges facing herbivores is the abrupt transition in food plant availability. For herbivores, space use and movement across ecotones is largely determined by the distribution of plants that allow acquisition of adequate nutrition, while minimizing exposure to toxic plant secondary compounds (PSCs; Dearing et al., 2000, 2005; Freeland & Janzen, 1974; Westoby, 1978). Mammalian herbivores have evolved numerous behavioral and physiological adaptations to maximize nutrition while minimizing toxin exposure including regulation of liver detoxification enzymes (Malenke et al., 2012), decrease in metabolic rate and physical activity when exposed to dietary PSCs (Sorensen et al., 2005b), and maintenance of a microbiome that facilitates nutrient acquisition and detoxification (Kohl et al., 2014). Mammalian herbivores may also diversify their diets to minimize overexposure to, or neutralize, toxins present (Iason & Villalba, 2006). Based on the degree to which mammalian herbivores modify their diets either spatially or temporally, they can be classified along a continuum of foraging strategies from generalist to specialist consumers (Shipley et al., 2009).

When mammals consume toxic plants they are not adapted to, they suffer energetic consequences that can lead to rapid weight loss and lowered body condition (Mangione et al., 2004; Sorensen et al., 2005a, 2005b). Given these consequences, we would expect mammalian herbivores to develop dietary preferences for plants with which they are familiar and which they can efficiently digest (Partridge, 1981). Hence, for herbivorous mammals, distinct vegetation communities across sharp ecotones may produce spatial variation in selection that leads to or reinforces distinct dietary preferences, which may in turn determine fine‐scale space use and a range of intra and interspecific interactions (Nosil et al., 2005; Via, 1999; Via et al., 2000).

**FIGURE 1:**
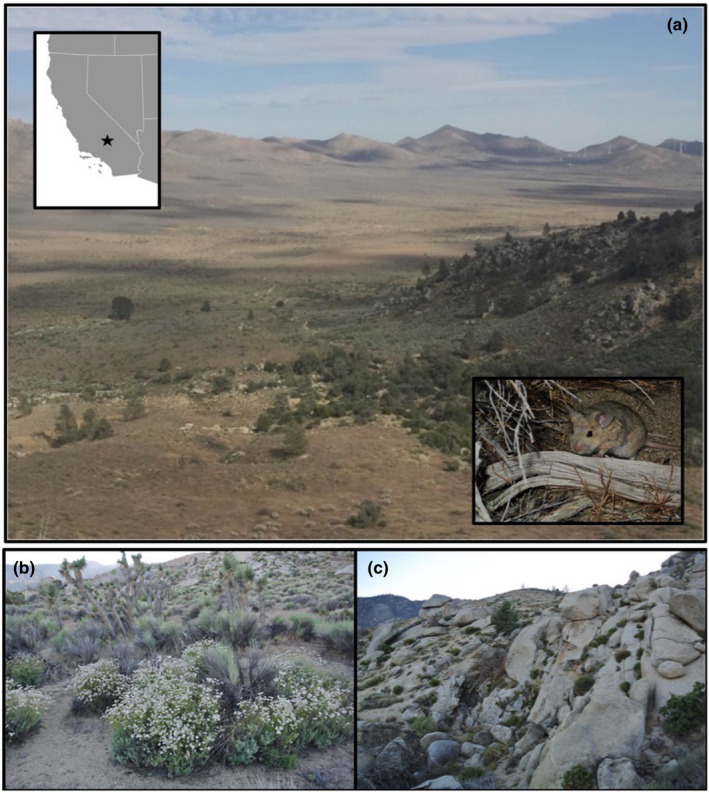
Panel (a) depicts the study site where the mesic hill transitions to the xeric flats. Photo taken from the north looking south. Black star in inset map represents approximate location of the study in Kelso Valley, California. Panels (b) and (c) depict habitat of the flats and hill habitats, respectively. Inset photo of woodrat is *Neotoma lepida*

One such ecotone exists on the western edge of the Kelso Valley, California where the southeastern slopes of the Sierra Nevada meet the valleys of the western Mojave Desert. Two closely related species of woodrat meet at this sharp ecotone: *Neotoma bryanti* (Bryant's woodrat) that primarily occur in the relatively mesic woodland and chaparral habitat of a rocky hill (hereafter called the “hill”), and *N. lepida* (Desert woodrat) that occur primarily in the adjacent Mojave Desert scrub habitat (hereafter called the “flats”; Shurtliff et al., 2014, Figure 2). The two species are estimated to have diverged ~1.6 mya based on mtDNA (Figure 1; Patton et al., 2007), and while they are largely spatially segregated between the two adjacent habitats, they do occasionally hybridize. These hybridization events lead to approximately 14% of individuals across the study site having hybrid ancestry, with backcrossing and introgression in both parental directions (Shurtliff et al., 2014; J. P. Jahner, T. L. Parchman, M. D. Matocq, unpublished data). Previous diet analyses (Matocq et al., 2020; Shurtliff et al., 2014) suggest that *N. bryanti* and *N. lepida* consume distinct diets in the hill and flats, respectively. As such, this system offers an opportunity to investigate dietary choices across a sharp ecotone, as well as the potential role of dietary differences in limiting interspecific contact and hybridization.

Here, we sought to further characterize the degree to which dietary composition and preference differ between pure *N. bryanti* and *N. lepida* in their respective native habitats, and to uncover the potential ecological correlates maintaining species differences in diet across this ecotone. We integrate both field and laboratory studies to ask the following questions: (1) Do *N. bryanti* and *N. lepida* maintain distinct diets across this sharp ecotone in both wet and dry seasons, and in wet and dry years? (2) Do these species consume plants in the wild in proportion to their availability in the habitat, or do they exhibit selection/preference for particular plants? (3) When given a choice in experimental trials, do woodrats exhibit the same dietary preferences as exhibited in field‐collected samples? (4) To what degree are plant nutritional content and plant secondary compounds correlated with dietary preferences? To address these questions, we quantify diet preferences in the wild using high‐throughput sequencing of the chloroplast *trn*L intron from woodrat fecal samples collected across the ecotone. We further examine these apparent patterns of preference by conducting an experimental choice trial. To understand the underlying drivers of fine‐scale diet differentiation in this system, we place these dietary preferences within the context of availability of these plants on the landscape, the plant secondary compound composition of these plants, and their nutritional quality. Our study provides a well‐developed example of fine‐scale diet differentiation in mammalian herbivores—differences across an ecotone that are maintained between the species in both wet and dry conditions.

## 2 MATERIALS AND METHODS

### 2.1 Study system

The study site is located in Kelso Valley, Kern Co., California, where *N. bryanti* and *N. lepida* meet and hybridize at the southern end of the Sierra Nevada mountain range (35°25′45N, 118°15′2W). The mesic “hill” habitat sharply transitions to the xeric “flats” habitat (Figure 1), and both parental species and hybrids can be found across a span of as little as tens of meters. The total area of the study site is approximately 50 hectares, approximately centered at the base of the hill (Figure 1). We conducted vegetation surveys in 27 plots (hill = 16, flats = 11) to estimate the abundance of the most common shrubs and trees (details in Supporting Information).

### 2.2 Woodrat species identity

We identified individuals as *N. bryanti* or *N. lepida* using microsatellite loci previously developed for these species (Sousa et al., 2007). For animals included in the preference trials (see below), we obtained ear biopsies from each individual and conducted DNA extraction, amplification and scoring of microsatellite loci as described in Coyner et al. (2015). We established species identity by conducting a Bayesian assignment test as implemented in STRUCTURE (Pritchard et al., 2000; Falush et al., 2003) at *K* = 2 as in Shurtliff et al. (2014) and used *q*_lepida_ values >90% to assign individuals to *N. lepida* and *q*_lepida_ values <10% to assign individuals to *N. bryanti*. To confirm the species identity of individuals included in the fecal metabarcoding, we used the same genotyping approach, but started with the gDNA extractions used for *trn*L sequencing (see below) and performed three replicate PCRs per sample.

### 2.3 Fecal metabarcoding

To determine the dietary composition of *N. bryanti* and *N. lepida* at our site, fecal samples were collected from 35 unique woodrat nests during March‐August of 2016. Species identity for fecal pellets was confirmed with microsatellite markers as described above. Clusters of approximately 10–20 fresh pellets were collected from *N. bryanti* nests in the hill habitat (*n* = 19) and *N. lepida* nests in the flats habitat (*n* = 16). These samples provided insight into diet in the spring months of March ‐ May (*N. bryanti = *11, *N. lepida* = 11) and summer months of July and August (*N. bryanti* = 8, *N. lepida* = 5). To ensure fecal pellets were characteristic of the sampling period, we located active latrines at woodrat nests and swept away all fecal material; after 3–4 nights, we collected fresh, adult‐sized fecal pellets. It is important to note that woodrat houses are solely occupied by one adult woodrat, and these animals are highly territorial, so there is limited chance that more than one woodrat contributed to the fresh fecal pellets we collected. We placed pellets into coin envelopes to dry, and stored them long‐term at −20°C. We submitted samples to Jonah Ventures LLC for sequencing of a portion of the chloroplast *trn*L intron to reconstruct relative summer diet composition (methods including extraction, PCR amplification, sequencing, and raw data processing in Supporting information). We removed operational taxonomic units (OTUs) that did not occur in at least one sample with more than 1% abundance. We confirmed identity of remaining OTUs by conducting a BLASTn search (https://blast.ncbi.nlm.nih.gov). The potential presence of a plant at the study site was determined based on our own field collections and the CalFlora database (https://www.calflora.org/). If a resulting search returned more than one possible species, genus, or family that might occur at the site, we report the highest level of taxonomy (i.e., genus, family). Finally, to confirm the *trn*L primers used would detect the most common plants at the site, and to generate known sequences (*i.e*. vouchers) for these plants, we sequenced the following collected at the study site: *Ericameria nauseosa*, *Artemisia tridentata*, *Eriogonum fasciculatum*, *Prunus fasciculata*, *Frangula californica*, and *Phacelia tanacetefolia*.

### 2.4 Diet composition

We used read counts of all identified plants to calculate Shannon diversity for diets of *N. bryanti* and *N. lepida* and performed a two‐sample *t*‐test in R to compare diversity in diet composition (R Core Team, 2016). We used read counts to determine if diets between the two species were distinct by performing a PERMANOVA using Bray–Curtis distances with the adonis function in the *vegan* package (Oksanen et al., 2013; R Core Team, 2016).

To estimate individual and population‐level (i.e., species) consumption of particular plants, we used both frequency of occurrence (FOO) and relative read abundance (RRA) of plant taxa identified in fecal samples. We considered a plant taxon present if it made up 1% or more of the total reads in a sample (Deagle et al., 2019). We calculated RRA for each plant within individual samples, and then averaged RRA values for *N. bryanti* and *N. lepida*. We used the signassoc function in the R package *indicspecies* (De Cáceres & Legendre, 2009) on the resulting presence/absence matrix and RRA datasets to determine which plants were significantly associated with either *N. bryanti* or *N. lepida*. Average RRA values have traditionally been viewed with caution as they are prone to recovery bias and other artifacts, but the information contained within read counts can still provide important insights into the relative importance of certain plants at the population level (Deagle et al., 2019). Previous authors have reported correlation between relative abundance of plants consumed and raw number of reads obtained (*r* ^2^ = .75, *p* < 10^–15^; Willerslev et al., 2014), and while FOO is less affected by recovery bias, RRA can provide a more accurate characterization of population‐level diet (Deagle et al., 2019). We sought to incorporate measures of presence/absence (FOO) and relative abundance (RRA) to characterize dietary differences at the population level in this study.

In order to take individual variation into account in estimates of population‐level consumption, we used a hierarchical Bayesian approach implemented in R using the *bayespref* package (Fordyce et al., 2011) to estimate population‐level consumption of the 5 most common plants identified in woodrat diets, which comprised ~80%–90% of total reads (Tables 1 and S1–S3). We pooled the remaining read counts from all other plant taxa into an “other” group. Rather than relying simply on RRA (as described above) to infer relative degree to which plants are consumed, this hierarchical Bayesian approach incorporates individual variation in our population‐level consumption estimates (Fordyce et al., 2011; Forister et al., 2013). We used raw read count data to run models. Raw read counts were not normally distributed, therefore we square‐root transformed read counts prior to analysis. We ran models for 50,000 iterations, with a burn‐in of 5,000 iterations and visually confirmed adequate chain‐mixing. Hereafter, we will refer to these estimates simply as consumption.

**TABLE 1:** Frequency of occurrence (FOO), relative read abundance (RRA), and where applicable, the percent abundance of woody plants in each habitat of plants identified in the diets of *N. bryanti* and *N. lepida*

Taxa identified	*N. bryanti* (*n* = 19)			*N. lepida* (*n* = 16)			*p*‐Value	
	FOO	RRA	%hill	FOO	RRA	%flats	FOO	RRA
*Prunus fasciculata*	0.21	0.04	0.04	1.00	0.79	0.10	**.01**	**.01**
*Frangula californica*	0.89	0.41	0.13	0.06	<0.01	0.01	**.01**	**.01**
*Phacelia tanacetefolia*	0.89	0.11		0.82	0.14		.65	.34
*Pinus spp*.	0.84	0.19	0.05	0.12	<0.01	<0.01	**.01**	**.01**
*Eriogonum umbellatum*	0.68	0.08		0.24	0.03		**.01**	.16
*Ribes amarum*	0.32	0.04	0.03	0.00	0.00	0	.06	**.01**
*Acmispon americanus*	0.32	0.03		0.00	0.00		.08	**.03**
*Asteraceae*	0.37	0.02		0.12	<0.01		.14	.17
*Ericameria nauseosa_voucher_*	0.21	<0.01	0.33	0.00	0.00	0.60	NA	NA
*Euphorbia maculata*	0.00	0.00		0.24	0.02		.10	.06
*Cercocarpus betuloides*	0.16	0.01	<0.01	0.00	0.00	0	.23	.29
*Salvia columbariae*	0.21	<0.01		0.00	0.00		.20	.10

Note

Here we include only those plants that occurred with FOO > 15% in spring and summer 2016 combined (full dietary plant list in Tables S1–S3). *p*‐Values are corrected for multiple comparisons. We confirmed the presence of *E. nauseosa* voucher sequences in some samples and therefore list FOO and RRA for those within the larger Asteraceae family. Bold indicates statistical significant *p* values.

Lastly, we considered diet composition of *N. bryanti* and *N. lepida* in this study (2016, a wet year) relative to that found previously (2013, a dry year; Matocq et al., 2020). We compare RRA values as consumption of plant food was not modeled for 2013 data.

### 2.5 Crude protein content of common shrubs

We characterized the nutritional value of common shrubs in each habitat and/or those that were most common in woodrat diets (see below) by measuring relative crude protein content. Crude protein content is considered the best single factor for determining nutritional value of forage plants (Sampson & Jesperson, 1963, pg. 20). We collected leaves and fresh green growth of *F. californica*, *P. fasciculata, E. nauseosa*, *A. tridentata*, and *E. fasciculatum* in summer and dried at ambient temperature. We estimated crude protein on the dry matter basis using the Kjeldahl method (Association of Official Analytical Chemists, 2002). In short, one gram of dried plant material was ground and digested in boric acid prior to titration to measure nitrogen content, which was multiplied by a factor of 6.25 (Association of Official Analytical Chemists, 2002).

### 2.6 Preference trials

We conducted preference trials in the field from Jun‐Aug of 2016 and 2017 to quantify dietary preference in *N. lepida* (*n* = 12; 3 F, 9 M) and *N. bryanti* (*n* = 15; 8 F, 7 M) for the two most common plants recovered from field diets (see below): *F. californica* and *P. fasciculata*. We provide all trapping and feeding trial details in the Supporting Information. All animal procedures were reviewed and approved by the University of Nevada Reno Institutional Animal Care and Use Committee, the California Department of Fish and Wildlife, and were consistent with the guidelines developed by the American Society of Mammalogists (Sikes et al., 2016).

We calculated a preference index with the following formula: Preference = (*p*−*f*)/*T*; where *p* is the total amount of *P. fasciculata* consumed during a trial, *f* is the total amount of *F. californica* consumed, and *T* is the total amount (grams) consumed. The resulting single response variable for preference during a given trial is bounded by −1 and +1; with positive values indicating preference for *P. fasciculata* and negative values indicating preference for *F. californica*. Results of a Shapiro–Wilk normality test conducted in R found these data to be normal (*W* = 0.95, *p* = .21). To test for confounding covariates, we used a linear model created in R with preference index as the response variable and species ID, and potentially confounding covariates (i.e., total time in trial, year, mixed vs. foliage food type, sex), as independent variables. This enabled us to rule out the possibility of confounding effects of these covariates on our independent variable of primary interest, species identity.

## 3 RESULTS

### 3.1 Vegetation community

The most common shrubs and trees on the hill were *E. nauseosa (*33%), *E. fasciculatum* (16%), *F. californica* (13%), *Ephedra* sp. (11%), *Hesperoyucca whipplei* (7%) and multiple species of *Pinus* (5%). The most common shrubs and trees in the flats were *E. nauseosa* (60%), *E. fasciculatum* (11%), *Yucca brevifolia* (11%), *P. fasciculata* (10%), and *A. tridentata* (6%). Relative proportions of all subshrubs, shrubs and trees are provided in the Supporting materials (Figure S1, Table S4). Vegetation diversity was greater on the hill (*H* = 1.50) than the flats (*H = *0.93; *t* = −4.40, *df* = 16.93, *p* < .001), and vegetation community composition differed between the hill and flats (MS = 1.83, *r* ^2^ = .33, *p* = .001). Of 91 woodrat nests in the flats, 59% were either directly at the base of *P. fasciculata* or were located in rocks with *P. fasciculata* adjacent, while the remaining were in *Y. brevifolia*, *E. nauseosa*, and *R. amarum*. Woodrat nests on the hill were primarily within large boulders with little if any immediately surrounding vegetation.

### 3.2 Diet composition, relative frequency of occurrence and relative read abundance

After filtering and verifying OTU representative sequences, we retained 847,690 reads from 35 woodrat fecal samples that represented 33 plant taxa (Tables 1 and S1–S3). During spring, diet diversity was greater in *N. bryanti* (*H* = 1.32) than in *N. lepida* (*H* = 0.71; *t* = 4.30, *df* = 19.87, *p* < .001), and diet composition was also distinct between *N. bryanti* and *N. lepida* (MS = 2.81, *r* ^2^ = .46, *p* = .001). During summer, diet diversity was also greater in *N. bryanti* (*H = *1.16) than in *N. lepida* (*H = *0.41; *t* = 5.51, *df* = 9.69, *p* < .001), and diet composition was also distinct between *N. bryanti* and *N. lepida* (MS = 1.99, *r* ^2^ = .60, *p* = .003). When data from both seasons were combined, diet diversity in *N. bryanti* (*H = *1.25), was twice that of *N. lepida* (*H = *0.62; *t* = 5.77, 32.92, *p* < .001), and diet composition was also distinct between the species (MS = 4.82, *r* ^2^ = .50, *p* = .001). In addition to the plants recovered from fecal samples, we confirmed that our primer set was able to recover the five common shrubs on which we tested them. Of note is that our known sequences for *Ericameria* and *Artemisia* are not different from many other species in the Asteraceae, thus all these similar sequences are collapsed into the Asteraceae (Tables 1 and S1–S3).

**FIGURE 2:**
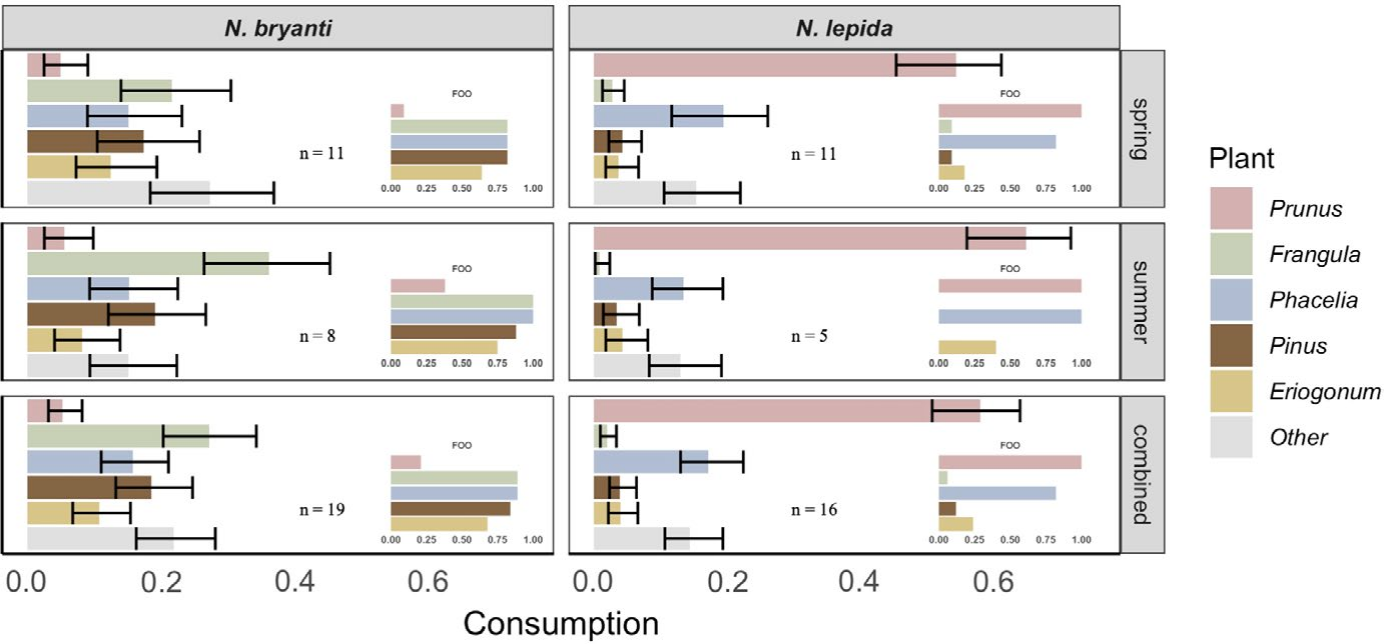
Consumption of the five most abundant plant taxa identified in woodrat diets in 2016 estimated for spring and summer individually, and both seasons combined. Consumption was estimated with *bayespref* using square root transformed read counts. Large bars are medians with 95% credible intervals from Bayesian posterior distributions. Insets represent frequency of occurrence (FOO) of these same plants

Overall, *N. bryanti* and *N. lepida* exhibit distinctly different diets, but do consume some of the same plants. The frequency of occurrence (FOO) and relative abundance (RRA) of all 33 plant taxa identified may be found in Tables 1 and S1–S3. Notably, *N. bryanti* exhibited a more diverse diet with *F. californica* as the most abundant food item in spring and summer combined (FOO = 0.89, RRA = 0.41; Table 1). *Pinus* spp. and *Phacelia tanacetefolia* also occurred in the diet of *N. bryanti* with greater than 80% FOO and over 10% RRA in spring and summer combined (Table 1). *Neotoma bryanti* increased consumption of *F. californica* in summer relative to spring evidenced by increases in both FOO and RRA (Figure 2, Tables S1 and S2). *Neotoma lepida* consumed a less diverse diet, with *P. fasciculata* being the most abundant in spring and summer (FOO = 1.00, RRA = 0.79; Table 1). *Neotoma lepida* increased consumption of *P. fasciculata* from spring to summer (RRA_spring_ = 0.74, RRA_summer_ = 0.91; Figure 2, Tables 1 and S1 and S2). Overall, RRA for the Asteraceae family did not exceed 2% for either *N. bryanti* or *N. lepida* and the overall frequency of occurrence was also low (FOO*_bryanti_* = 0.37, FOO*_lepida_* = 0.12). Thus we are confident that, even with our inability to discriminate within the Asteraceae family, woodrats consume very little if any *E. nauseosa* or *A. tridentata* at our site.

Results of our hierarchical Bayesian modeling were consistent with diet composition based on FOO and RRA estimates. Notably, estimates of consumption using *bayespref* were less extreme than those from average RRA values (Tables 1 and S1–S3, S5, Figure 2). *Frangula californica* was still the most common single plant in the diet of *Neotoma bryanti* and increased from spring to summer (consumption_spring_ = 0.22 [95% CI 0.14–0.30], consumption_summer_ = 0.36 [95% CI 0.26–0.45]; Table S5, Figure 2). More than half the diet of *N. lepida* was composed of *P. fasciculata* also increased from spring to summer (consumption_spring_ = 0.54 [95% CI 0.45–0.61], consumption_summer_ = 0.65 [95% CI 0.56–0.71]; Table S5, Figure 2). While diets of *N. bryanti* and *N. lepida* were vastly different, *Phacelia tanacetefolia,* an annual forb, was found to make up ~13%–19% of the diet of both species (Table S5, Figure 2).

Our measures of diet composition in this study were largely consistent with those previously described in Matocq et al. (2020). Their measure of diet occurred during the summer of 2013, an extreme drought year, wherein *Neotoma bryanti* consumed a high level of *F. californica* (RRA = 0.52) and *N. lepida* consumed large amounts of *P. fasciculata* (RRA = 0.59; Matocq et al., 2020). During spring of a wet year (2016; this study), when more vegetation diversity was available, *N. bryanti* reduced consumption of the “difficult” *F. californica* relative to summer (RRA_spring_ = 0.35, RRA_summer_ = 0.51). In contrast, *N. lepida* maintained high levels of *P. fasciculata* in its diet whether an extreme drought year summer (see above) or a wet‐year summer (i.e., 2016, RRA_summer_ = 0.91. Even during a “superbloom” spring, arguably the highest diversity this site experiences, *N. lepida* still consumed high quantities of *P. fasciculata* (RRA_spring_ = 0.74).

### 3.3 Crude protein content

*Prunus fasciculata* and *F. californica* had among the highest levels of summer crude protein content, 15.1% and 12.4% respectively (Table 2). Our measurements of crude protein for *E. nauseosa, A. tridentata,* and *E. fasciculatum* were 8.0, 8.4, and 5.1, respectively. Sampson and Jesperson (1963) reported average crude protein content of *F. californica* leaves as high as 19% from April to August. Summer crude protein content of *A. tridentata* was reported at 9.9% during August, with values as high as 15% during spring (Sampson & Jesperson, 1963; Welch, 1989). Crude protein content of *E. nauseosa* can range from a minimum of 9% to a high of 11.8% when new growth forms (Sampson & Jesperson, 1963). Crude protein content of *E. fasciculatum* varied from 5.4% in summer to 8.6% for new growth (Genin & Badan‐Dangon, 1991). We were unable to find reported crude protein content of *P. fasciculata* in the literature.

**TABLE 2:** Crude protein content (percent dry matter basis) of five common perennial shrubs found at the study site

Species	Crude protein this study	Crude Protein Literature	Reference
*Artemisia tridentata*	8.4 ± 0.9	8.5–15	Welch (1989), Sampson and Jesperson (1963), Cook and Harris (1950), Kelsey et al. (1982)
*Ericameria nauseosa*	8.0 ± 1.4	7.8–11.8	Welch (1989), Sampson and Jesperson (1963)
*Eriogonum fasciculatum*	5.1 ± 1.1	5.1–5.7	Genin and Badan‐Dangon (1991)
*Prunus fasciculata*	15.1 ± 0.1	N/A	No published record
*Frangula californica*	12.4 ± 0.2	7.5–19	Sampson and Jesperson (1963)

### 3.4 Preference trials

**FIGURE 3:**
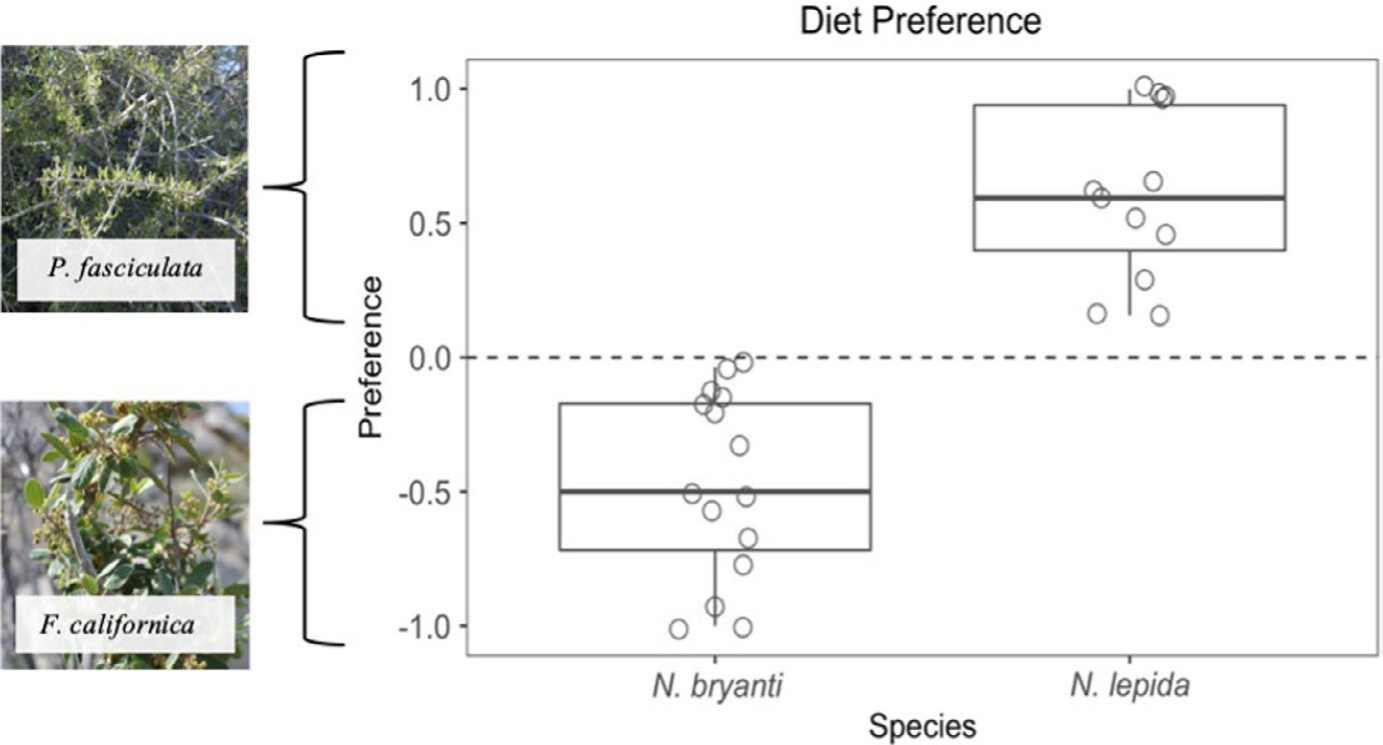
Group level average preference index for each species; *N. bryanti* and *N. lepida* along the *x*‐axis. Preference as measured here is an index of the amount of *P. fasciculata* minus the amount of *F. californica* consumed divided by the total amount of food consumed during the trial. The *y*‐axis represents this index: positive values indicate preference for *Prunus fasciculata* and negative values indicate preference for *Frangula californica*

A total of 27 individuals were included in diet trials: *N. bryanti* (*n* = 15), *N. lepida* (*n* = 12). We found that preference was significantly different between species (*p* < .001, Table 3). *N. bryanti* exhibited a preference for *F. californica* (preference = −0.47 [95% CI −0.66 to −0.28], while *Neotoma lepida* preferred *P. fasciculata* (preference = 0.61 [95% CI 0.41–0.81]; Figure 3). There was variation in preference index among individuals. However, all *N. lepida* individuals showed preference for *P. fasciculata* with two individuals consuming only that plant, and all *N. bryanti* individuals showed preference for *F. californica* with two individuals consuming only that plant.

**TABLE 3:** Effects of variables included in linear model of preference trials

Variable	Estimate	*SD*	*t* Value	*p*‐Value
(Intercept)	−0.19333	0.17713	−1.091	.2880
*N. lepida*	1.17135	0.14086	8.316	**<.001**
Sex	−0.13788	0.13989	−0.986	.3361
Mass change in trial	−0.16515	0.08127	−2.032	.0556
Year	−0.50832	0.31591	−1.609	.1233
Duration of Trial	−0.17816	0.16896	−1.054	.3042
Food Type	0.04444	0.20654	0.215	.8318

Note

The dependent variable was the preference index for either *F. californica* or *P. fasciculata*—measured as the amount of *P. fasciculata* minus the amount of *F. californica* consumed divided by the total amount of food consumed during the trial. Bold indicates statistical significant *p* values.

*Results of overall model*: Residual standard error: 0.3225 on 20 degrees of freedom; multiple *R*‐squared: .8023; adjusted *R*‐squared: .743; *F*‐statistic: 13.53 on 6 and 20 degrees of freedom; *p*‐value: <.001.

## 4 DISCUSSION

Despite ongoing hybridization between *N. bryanti* and *N. lepida* (Shurtliff et al., 2014), we found differences in dietary preference and dietary composition between these two species; differences that were maintained in both wet and dry years, and across seasons. The primary plants differentially preferred by each species are nutritious relative to other available plants, but also potentially toxic in unique ways, suggesting these species may have evolved or developed distinct metabolic strategies to reduce toxin exposure. Given the degree of dietary plasticity we observed across seasons in natural diets and in preference trials, we find that *N. bryanti* is more of a dietary generalist than *N. lepida*. Dietary differences between the species likely contribute to their spatial segregation across the ecotone, which ultimately determines their opportunities for interspecific interactions, including hybridization.

At this ecotone, the vegetation of the hill community is more diverse and largely distinct from that of the flats, and this diversity and differentiation is partly reflected in the diets of the woodrats that occupy these habitats (Figure 2 and Figure S1, Tables 1 and S1–S3). Overall, dietary diversity of *N. bryanti* on the hill was twice that of *N. lepida* individuals living in the flats. Despite the diversity of plants consumed by *N. bryanti*, *F. californica* appears to predominate their diet. In contrast, *N. lepida* in the flats have a diet dominated by *P*. *fasciculata*. For both *N. bryanti* and *N. lepida*, these food plants (i.e., *F. californica* and *P. fasciculata*) were consumed at higher rates than their availability on the landscape, suggesting dietary selection (Hodgson, 1979).

During spring and summer of 2016, we show that the diet of *N. bryanti* on the hill is dominated by *F. californica* while the diet of *N. lepida* on the flats is dominated by *P. fasciculata*. However, we did find that some *N. bryanti* on the hill consumed a small amount of *P. fasciculata,* while *N. lepida* on the flats infrequently consumed *F. californica*. This result from our sample of wild diets is at least partly due to the relative rarity of these two plants in the “alternate” habitat. However, results of our 2‐choice trial show that even when given a choice of both plants, on average, *N. bryanti* primarily consumed *F. californica* and *N. lepida* primarily consumed *P. fasciculata*. As such, on average, individuals in our experimental trial showed a preference for the plant they most commonly consume in the natural environment. Overall, our field and experimental results demonstrate that *N. bryanti* show a preference for *F. californica* and *N. lepida* show a preference for *P. fasciculata*, which may reflect differences in behavioral acclimation to different resources and/or underlying species differences in their ability to metabolize these particular plants.

Despite the overall preference *N. bryanti* and *N. lepida* exhibit for these plants, there was a great deal of individual variation in our experimental trials. Specifically, most individuals consumed at least some of the presumably novel plant. This is a foraging behavior animals may employ to identify new food resources (Partridge, 1981), and one we might expect when individuals are exposed to novel food items. This short‐term consumption of a potentially novel, chemically distinct plant did not appear to have negative consequences for experimental animals as none lost excessive weight over this short period (i.e. >10% body mass) and animals remained alert and responsive. Overall, *N. bryanti* showed less extreme preference than *N. lepida* and these results are consistent with several *N. bryanti* on the hill consuming *P. fasciculata*, albeit in very low amounts, while *N. lepida* on the flats rarely consume *F. californica*. Both of these lines of evidence suggest that *N. bryanti* may be further towards the generalist end of the spectrum of specialization, while *N. lepida* may be further towards the specialist end of the spectrum (Shipley et al., 2009).

Herbivores may employ a range of dietary strategies, from specialist to generalist, to balance nutrient acquisition and exposure to plant PSCs. Specifically, facultative specialists exhibit diets largely restricted to a single “difficult” (i.e., potentially toxic) food item, but are capable of expanding their diet when resource availability allows. In contrast, facultative generalists typically maintain more diverse diets, but are capable of restricting their diets to a “difficult” plant when environmental conditions limit food resources (Shipley et al., 2009). *Frangula californica* and *P. fasciculata* are known to contain PSC’s that deter herbivory. *Frangula californica* contains anthraquinones, that can cause severe damage to the intestinal lining of mammals, and have hepatotoxic effects (Jung et al., 2011; Qin et al., 2016). In contrast, *P. fasciculata* contains cyanogenic glycosides, also highly toxic once cyanide is released from the parent compound (Vetter, 2000). Chemical analysis of plants from the Kelso Valley have shown that *F. californica* and *P. fasciculata* contain chemical peaks consistent with the anthraquinone, emodin, in *F. californica* and the cyanogenic glycoside, prunasin, in *P. fasciculata* (Matocq et al., 2020). Given the potential toxicity of these plants, why do woodrats eat so much of them? On one hand, from a chemical perspective, these plant species may be among the best of a bad lot. The other common shrubs present—*E. nauseosa, A. tridentata*, and *Ephedra* are also known to be chemically well‐defended and/or energetically costly to consume (Dial, 1988; Halls et al., 1994; Johnson et al., 1976). In addition to this, though, our nutritional analyses coupled with information available in the literature suggest that *F. californica* and *P. fasciculata* are among the most nutritious plants at this site in terms of crude protein. The composition of woodrat diets is likely a result of how *N. bryanti* and *N. lepida* have come to balance access to nutrition while minimizing their overexposure to plant secondary compounds, as seen in other small mammals (Ulappa et al., 2014).

The degree to which woodrats and other herbivores can minimize their exposure to toxins by diversifying their diets (Freeland & Janzen, 1974) depends on environmental conditions and associated plant availability. For this study site, we can begin to assess dietary plasticity under different seasonal and annual conditions by combining current results with data collected in previous years (Matocq et al., 2020). At one extreme is the 2013 snapshot of diet composition, which was taken in summer of an extreme drought wherein California received less precipitation than in any previous year in the 119‐year observational record (Swain et al., 2014), and few annual forbs were observed at the site (M. D. Matocq, personal observation). This is in contrast to conditions at the site during 2016 ‐ a wet and warm year facilitated by El Niño conditions that led to a spectacular 2016 spring “superbloom” event (Treonis et al., 2019) characterized by high annual forb diversity across the Mojave desert. These snapshots of diet composition (i.e., 2013 and 2016) capture aridity extremes from centennial‐scale drought, to wet‐year summer, to wet‐year spring, and thus, a plant diversity/availability gradient from low to high for this site. As expected, if *N. bryanti* is a facultative generalist, high plant diversity in spring 2016 led to a decrease in consumption of *Frangula* whereas, *N. lepida* maintained high consumption of *Prunus*, regardless of availability of spring forbs. Others have classified *N. lepida* as a facultative specialist (Dial, 1988; Shipley et al., 2009; Skopec et al., 2015), and our data support this classification. Indeed, *N. lepida* can consume large quantities of what is considered to be a potentially toxic plant. Although *N. lepida* is capable of consuming other plants at this site, individuals appear to prefer *P. fasciculata* even when other options are available, suggesting local specialization on this plant. *Neotoma bryanti* is also capable of consuming large quantities of a potentially toxic plant, *F. californica*. Yet, when given the option, we observed that *N. bryanti* will diversify its diet while still maintaining a high proportion of the “difficult” plant in its diet—further supporting *N. bryanti* at this site as a facultative generalist. It should be noted that any study of diets in wild rodents that cache or hoard, as woodrats do, cannot discriminate between items that were eaten fresh versus those eaten after storage. Caching may reduce toxin content in plants, especially those with volatile compounds (i.e., *Juniperus*, Torregrossa & Dearing, 2009). While the primary compounds in *Frangula* and *Prunus* are not volatile, we do not know how these compounds would degrade over time if stored. Likewise, we do not know the extent to which woodrats at this site cache these plants.

Another critical ecological driver of diet composition and breadth at this study site is simply the presence of a closely related congener. Specifically, the narrower dietary niche of *N. lepida* at this site could in part be driven by competition with *N. bryanti*. Shurtliff et al., (2013) showed in laboratory trials that the relatively large‐bodied *N. bryanti* is more aggressive than the relatively small‐bodied *N. lepida*. Interspecific competition is thought to be an important driver of dietary differentiation and fine‐scale space use in interspecific contact zones between woodrats, with the large‐bodied species typically monopolizing optimal nest sites (Cameron, 1971; Dial, 1988). *Neotoma bryanti* at this site monopolize what is likely the more optimal, relatively thermally stable boulder nesting area of the hill (Brown, 1968). We suspect inherent differences in behavioral, physical, and metabolic capabilities have allowed *N. bryanti* to monopolize the hill habitat with its diversity of dietary plants, while *N. lepida* have persisted at the site in part because of its ability to locally specialize on *P. fasciculata*.

Woodrats are well‐known for their capacity to consume large quantities of potentially toxic plants (*Larrea tridentata*—Mangione et al., 2000; *Juniperu*s sp.—Dial, 1988, Skopec et al., 2007). In particular, *N. lepida* is known to locally specialize on chemically distinct plants across its range (*Larrea tridentata*—Mangione et al., 2000; *Juniperus*—Stones and Hayward (1968), and here, *P. fasciculata*). The mechanisms that underlie a woodrat's capacity to detoxify these diets likely include expression of their own detoxifying enzymes (Kitanovic et al., 2018; Malenke et al., 2012) and the activity of their gut microbiota (Kohl et al., 2014). Studies are needed to identify loci that are responsible for detoxification of different compounds, the degree to which specific alleles or pathways effectively metabolize particular PSC’s, and the interaction between mammalian and microbial genomes in creating toxin resistant phenotypes (Forbey et al., 2018). If unique metabolic adaptations or microbial combinations allow *N. bryanti* and *N. lepida* to metabolize different plants in their respective habitats, then migrant and hybrid individuals that do not possess habitat‐specific genomic or microbial combinations may suffer reduced fitness (Nosil et al., 2005; Via, 1999; Via et al., 2000). Selection against migrants would minimize opportunities for interspecific contact and mating (prezygotic isolation), while selection against hybrids with suboptimal allelic or microbial combinations would further limit introgression between the species (postzygotic isolation). Continued integration of field and laboratory studies will be needed to identify the mechanisms that underlie metabolic processing of these diets, and how diet‐related selection is influencing the evolutionary trajectory of these species.

## CONFLICT OF INTEREST

The authors declare no conflicts of interest.

## AUTHOR CONTRIBUTION

**Danny P. Nielsen:** Formal analysis (lead); Writing‐original draft (equal); Writing‐review & editing (equal). **Marjorie D. Matocq:** Conceptualization (lead); Funding acquisition (lead); Project administration (lead); Writing‐original draft (equal); Writing‐review & editing (equal).

## Supporting information

Supplementary Material

## Data Availability

Raw sequences from this study are available in the NCBI Sequence Read Archive under the BioProject accession number PRJNA700875. The remaining data are available from the Dryad Digital Repository: https://doi.org/10.5061/dryad.8sf7m0cmc.
