# Supplementary material for: Differences in dietary composition and preference maintained despite gene flow across a woodrat hybrid zone
Source: Ecol Evol. 2021 Mar 17;11(9):4909–19. doi: 10.1002/ece3.7399 (PMC8093690; doi:10.1002/ece3.7399)
Supplement: Supplementary file 1 — Supplementary Material [file ECE3-11-4909-s001.docx]

**Supporting Information**

**Text S1: Relative proportion of woody shrubs and trees**

To quantify the perennial dietary landscape available to woodrats, we measured the relative proportion of woody shrubs and trees within a 25 m radius plot centered on active woodrat nests in both the hill (n = 16) and flats (n = 11) habitat. Nests were located and 25 meter transect lines were run from the center in each cardinal direction to delineate the plot. We identified and counted all shrubs and trees in each plot and estimated the relative number of individuals. We calculated Shannon diversity for each plot and performed a two-sample t test to compare diversity in woody shrubs and trees between the hill and flats (R Core Team 2016). To determine if the two habitats were characterized by distinct vegetation communities, we performed a PERMANOVA using the Bray-Curtis distances and with the adonis function in the *vegan* package (Oksanen et al. 2013). Because most *N. lepida* nests in the flats habitat occurred at the base of shrubs, we noted the species of plant in which nests were constructed. Average relative proportions were calculated for 15 common shrubs and trees occurring across the study site (Fig. S1, Table S4).

**Text S2: Methods for Fecal Metabarcoding**

Fecal samples were submitted in barcode-labeled vials to Jonah Ventures LLC (Boulder, CO) for *trn*L sequencing. Samples were assigned to a well in a 96 well plate. Then, a sterile cotton swab was dipped in nuclease free water before swabbing the sample. Approximately 0.25 grams of each sample was used for DNA extractions. Sample swabs were placed in corresponding well with sterile tweezers. Samples were then processed or stored in -20°C until the extraction process could be performed.

Genomic DNA was extracted using the DNeasy PowerSoil HTP 96 Kit (Cat # 12955-4) according to the manufacturer’s instructions. For each sample, a portion of the chloroplast *trn*L intron was PCR amplified using the c and h *trn*L primers (Taberlet et al. 2007):, *c –* CGAAATCGGTAGACGCTACG and *h* – CCATTGAGTCTCTGCACCTATC. Primers also had a 5’ adaptor sequence for indexing and Illumina sequencing. Each 25µL PCR included 0.4 µM of each primer and 1µl of gDNA (Promega catalog # M5133, Madison, WI). DNA was PCR amplified with the following: initial denaturation at 94°C for 3 minutes, followed by 40 cycles of 30 seconds at 94°C, 30 seconds at 55°C, and 1 minute at 72°C, and a final elongation at 72°C for 10 minutes.

Amplicons were cleaned with UltraClean 96 PCR Cleanup Kit (384) (cat#12596-4) (MoBio) according to manufacturer’s instructions. Another round of PCR was used to give samples unique index sequences. Successful barcode attachment was confirmed on 2% agarose gel. Final amplicons from each sample were cleaned and normalized using SequalPrep Normalization Plates (Life Technologies, Carlsbad, CA) according to manufacturer’s instructions; samples were pooled and sequenced on an Illumina MiSeq (San Diego, CA) in the CU Boulder BioFrontiers Sequencing Center.

Sequences were demultiplexed using Golay barcodes via QIIME v1.9.1 (Caporaso et al. 2010). Forward and reverse read sequences were trimmed to 235 nucleotides using the option -fastx_truncate, and then merged with the -fastq_mergepairs option in usearch8 (Edgar 2010). Primers were removed with cutadapt (Martin 2011) and *trn*L amplicons were processed using the UPARSE pipeline (Edgar 2013). Plant taxonomy was assigned with the SINTAX protocol (http://www.drive5.com/usearch/manual/utax_user_train.html) available in usearch (v8.1.1861; Edgar 2010). Sequences were quality trimmed and OTUs were clustered at 99% similarity with de novo chimera checking. A custom SINTAX *trn*L reference database was constructed by downloading any annotated GenBank (Benson et al. 2005) records that contain the *trn*L gene. This was then used to assign potential taxonomic groups. All extracted amplicon regions were dereplicated to 100% sequence identity and any identical sequence across lineages were collapsed to the lowest-common-ancestor. Closed-reference OTUs were generated by searching against the *trn*L reference database at 99% sequence similarity. Additional OTUs were generated from plant voucher specimens that were collected by D. Nielsen from the study site. Finally, representative sequences that were present with at least 1% abundance were manually blasted in GenBank to confirm taxonomic identification of plants known to be present at the study site (resulting plant IDs in Tables S1-S3).

**Text S3: Methods for Experimental Preference Trials**

To capture live animals for preference trials, we placed two tomahawk live traps wherever a nest or sign of woodrat activity was present. Trap treadles were scented with a very small amount of peanut butter and oats in order to lure woodrats, but not enough to be consumed and potentially alter fecal composition or microbiome. Each new animal trapped was given a unique ear tag for identification, sexed, weighed, measured, and a portion of the ear pinnae was removed with sterilized surgical scissors and stored in 95% ethanol at ambient temperature for genotyping. Animals used in preference trials were transported in tomahawk traps back to the temporary field lab, were provided with cotton bedding, water, and were placed under dark cover for the remainder of the day. Woodrats were fasted for at least 8 hours prior to trials. We included only adult individuals, and females showed no sign of reproductive activity.

Enclosures for preference trials were designed from plastic containers with lids that allowed air flow (60.3cm L x 40.6 W x 34.3 H). Fresh cotton was provided during each trial for bedding material. Because fresh vegetation was provided from known diets at this site, water was not provided after we confirmed that woodrats did not readily consume freely available water. Clippings of *F. californica* and *P. fasciculata* were collected haphazardly from the study site and provided in equal amount by weight to each individual woodrat during each trial. For *P. fasciculata*, clippings included both leaves and freshly grown stems as bark of *P. fasciculata* is consumed by woodrats as well (personal obs. D. Nielsen). Fruits of *F. californica* were used for some of the 2016 trials because they were available at the site. All other trials consisted of only foliage from each plant species. We recorded plant type as mixed (fruits and foliage), and foliage (foliage only) for each trial. We conducted preference trials at night between the hours of 2000-0500; the active hours of these nocturnal herbivores. Researchers conducting cafeteria-style preference tests with other species of *Neotoma* have reported detecting dietary preference within only 30 minutes (McEachern et al. 2006). As woodrats are known to be novelty seekers, we allowed trials to run between 4 and 8 hours, or as long as they exhibited foraging behavior, to allow sufficient time for woodrats to exhibit feeding on ‘novel’ plants as well as preferred plants. We monitored woodrats and removed them from the trial as soon as activity and feeding had ceased. No woodrat completely consumed all of either plant material provided.

We accounted for evaporative water loss of plant material during trials using control enclosures maintained during each trial. After each trial (experimental or control), the remaining material was removed and weighed to the nearest 0.1 gram using a digital scale to calculate the amount of plant mass lost during the trial. For each trial night, we corrected the amount consumed by woodrats with the amount of change in mass of each plant in control enclosures. This allowed us to account for evaporative change in plant mass and assign a conservative consumption value to each plant type during each trial. We did not include trials in which woodrats consumed less than 1 gram of food material total. At the end of each trial, animals were re-weighed and released back at the point of capture. Animals were kept no longer than ~ 24 hours before being released.

**Literature Cited**

Benson, D. A., Karsch-Mizrachi, I., Lipman, D. J., Ostell, J., & Wheeler, D. L. (2005). GenBank. *Nucleic acids research*, *33*(suppl_1), D34-D38.

Caporaso, J. G., Kuczynski, J., Stombaugh, J., Bittinger, K., Bushman, F. D., Costello, E. K., ... & Huttley, G. A. (2010). QIIME allows analysis of high-throughput community sequencing data. *Nature methods*, *7*(5), 335-336.

Edgar, R. C. (2010). Search and clustering orders of magnitude faster than BLAST. *Bioinformatics*, *26*(19), 2460-2461.

Edgar, R. C. (2013). UPARSE: highly accurate OTU sequences from microbial amplicon reads. *Nature methods*, *10*(10), 996.

Martin, M. (2011). Cutadapt removes adapter sequences from high-throughput sequencing reads. *EMBnet. journal*, *17*(1), 10-12.

McEachern, M.B., A Eagles‐Smith, C., M Efferson, C., & H Van Vuren, D. (2006). Evidence for local specialization in a generalist mammalian herbivore, *Neotoma fuscipes.* *Oikos*, *113*(3), 440-448.

Oksanen, J., Blanchet, F. G., Kindt, R., Legendre, P., Minchin, P. R., O’hara, R. B., ... & Oksanen, M. J. (2013). Package ‘vegan’. *Community ecology package, version*, *2*(9), 1-295.

R Core Team (2016). R: A language and environment for statistical computing. R Foundation for Statistical Computing, Vienna, Austria. URL <https://www.R-project.org/>.

Taberlet P., Coissac E, Pompanon F, et al. (2007) Power and limitations of the chloroplast trnL (UAA) intron for plant DNA barcoding. *Nucleic Acids Research*, 35(3), e14-e14 doi:10.1093/nar/gkl938.

| **Taxa Identified** | ***N. bryanti* (n = 11)** | | |  | ***N. lepida* (n = 11)** | | | |  |  | ***P-value*** | |
| --- | --- | --- | --- | --- | --- | --- | --- | --- | --- | --- | --- | --- |
|  | | FOO | RRA | %hill | FOO | RRA | | %flats | |  | FOO | RRA |
| *Prunus fasciculata* | | 0.09 | 0.04 | 0.04 | 1.00 | 0.74 | 0.10 | | |  | 0.01 | 0.01 |
| *Frangula californica* | | 0.82 | 0.35 | 0.13 | 0.09 | <0.01 | 0.01 | | |  | 0.01 | 0.01 |
| *Phacelia tanacetefolia* | | 0.82 | 0.12 |  | 0.82 | 0.18 |  | | |  | 0.88 | 0.33 |
| *Pinus* | | 0.82 | 0.18 | 0.05 | 0.09 | <0.01 | <0.01 | | |  | 0.01 | 0.01 |
| *Eriogonum umbellatum* | | 0.64 | 0.10 |  | 0.18 | 0.03 |  | | |  | 0.05 | 0.34 |
| *Ribes amarum* | | 0.45 | 0.05 | 0.03 | 0.00 | 0.00 | 0 | | |  | 0.04 | 0.03 |
| *Acmispon aericanus* | | 0.55 | 0.05 |  | 0.00 | 0.00 |  | | |  | 0.02 | 0.03 |
| *Asteraceae* | | 0.45 | 0.01 |  | 0.18 | <0.01 |  | | |  | 0.38 | 0.23 |
| *Ericameria nauseosa_voucher_* | | 0.27 | <0.01 | 0.33 | 0.00 | 0.00 | 0.60 | | |  | NA | NA |
| *Euphorbia maculata* | | 0.00 | 0.00 |  | 0.18 | 0.02 |  | | |  | 0.43 | 0.23 |
| *Cercocarpus betuloides* | | 0.18 | <0.01 | <0.01 | 0.00 | 0.00 | 0 | | |  | 0.44 | 0.42 |
| *Mentzelia nitens* | | 0.18 | 0.01 |  | 0.09 | <0.01 |  | | |  | 0.71 | 0.63 |
| *Salvia columbariae* | | 0.36 | 0.02 |  | 0.00 | 0.00 |  | | |  | 0.13 | 0.05 |
| *Eriastrum densifolium* | | 0.09 | 0.01 |  | 0.00 | 0.00 |  | | |  | 0.76 | 0.14 |
| *Populus* | | 0.09 | 0.01 |  | 0.00 | 0.00 |  | | |  | 0.72 | 0.68 |
| *Yucca brevifolia* | | 0.00 | 0.00 | 0.01 | 0.00 | 0.00 | 0.11 | | |  | 1.00 | 0.02 |
| *Leptosyne* | | 0.18 | <0.01 |  | 0.00 | 0.00 |  | | |  | 0.37 | 0.20 |
| *Ephedra* | | 0.09 | <0.01 | 0.11 | 0.00 | 0.00 | <0.01 | | |  | 0.74 | 0.01 |
| *Chenopodium* | | 0.00 | 0.00 |  | 0.09 | <0.01 |  | | |  | 0.73 | 0.37 |
| *Camissonia campestris* | | 0.00 | 0.00 |  | 0.09 | <0.01 |  | | |  | 0.70 | 0.26 |
| *Stephanomeria* | | 0.09 | <0.01 |  | 0.00 | 0.00 |  | | |  | 0.72 | 0.13 |
| *Descurainia pinnata* | | 0.00 | 0.00 |  | 0.09 | <0.01 |  | | |  | 0.68 | 0.08 |
| *Cuppressaceae* | | 0.09 | <0.01 |  | 0.00 | 0.00 |  | | |  | 0.73 | 0.77 |
| *Cirsium arvense* | | 0.00 | 0.00 |  | 0.00 | 0.00 |  | | |  | 1.00 | 0.44 |
| *Erodium cicutarium* | | 0.00 | 0.00 |  | 0.09 | <0.01 |  | | |  | 0.68 | 0.05 |
| *Claytonia sp* | | 0.18 | <0.01 |  | 0.00 | 0.00 |  | | |  | 0.40 | 0.02 |
| *Poaceae* | | 0.00 | 0.00 |  | 0.09 | <0.01 |  | | |  | 0.70 | 0.28 |
| *Lupinus* | | 0.09 | <0.01 |  | 0.00 | 0.00 |  | | |  | 0.75 | 0.06 |
| *Ceanothus cordulatus* | | 0.00 | 0.00 |  | 0.00 | 0.00 |  | | |  | 1.00 | 1.00 |
| *Phragmites australis* | | 0.00 | 0.00 |  | 0.09 | <0.01 |  | | |  | 0.70 | 0.12 |
| *Camissonia kernensis* | | 0.00 | 0.00 |  | 0.09 | <0.01 |  | | |  | 0.74 | 0.15 |
| *Thysanocarpus* | | 0.09 | <0.01 |  | 0.00 | 0.00 |  | | |  | 0.72 | 0.68 |
| *Scrophularia desertorum* | | 0.00 | 0.00 |  | 0.00 | 0.00 |  | | |  | 1.00 | 0.25 |
| *Bromus* | | 0.00 | 0.00 |  | 0.09 | <0.01 |  | | |  | 0.70 | 0.25 |

**Table S1**: Spring 2016 frequency of occurrence (FOO) and relative read abundance (RRA) of 33 unique plant taxa identified in the diets of *N. bryanti* and *N. lepida*. *P-*values are corrected for multiple comparisons. Where applicable, percent abundance of woody plants in each habitat is reported.

**Table S2**: Summer 2016 frequency of occurrence (FOO) and relative read abundance (RRA) of 33 unique plant taxa identified in the diets of *N. bryanti* and *N. lepida*. *P-*values are corrected for multiple comparisons. Where applicable, the percent abundance of woody plants in each habitat is reported.

| **Taxa Identified** | ***N. bryanti* (n = 8)** | | |  | ***N. lepida* (n = 5)** | | |  | ***P-value*** | |
| --- | --- | --- | --- | --- | --- | --- | --- | --- | --- | --- |
|  | FOO | RRA | %hill | | FOO | RRA | %flats | | FOO | RRA |
| *Prunus fasciculata* | 0.38 | 0.05 | 0.04 | | 1.00 | 0.91 | 0.10 | | 0.10 | 0.01 |
| *Frangula californica* | 1.00 | 0.51 | 0.13 | | 0.00 | 0.00 | 0.01 | | 0.01 | 0.01 |
| *Phacelia tanacetefolia* | 1.00 | 0.08 |  | | 1.00 | 0.03 |  | | 1.00 | 0.39 |
| *Pinus* | 0.88 | 0.20 | 0.05 | | 0.00 | 0.00 | <0.01 | | 0.01 | 0.01 |
| *Eriogonum umbellatum* | 0.75 | 0.06 |  | | 0.40 | 0.01 |  | | 0.38 | 0.09 |
| *Ribes amarum* | 0.13 | 0.03 | 0.03 | | 0.00 | 0.00 | 0 | | 0.82 | 0.06 |
| *Acmispon americanus* | 0.00 | 0.00 |  | | 0.00 | 0.00 |  | | 1.00 | 0.34 |
| *Asteraceae* | 0.25 | 0.03 |  | | 0.00 | 0.00 |  | | 0.62 | 0.60 |
| *Ericameria nauseosa_voucher_* | 0.13 | <0.01 | 0.33 | | 0.00 | 0.00 | 0.60 | | NA | NA |
| *Euphorbia maculata* | 0.00 | 0.00 |  | | 0.40 | 0.02 |  | | 0.27 | 0.20 |
| *Cercocarpus betuloides* | 0.13 | 0.02 | <0.01 | | 0.00 | 0.00 | 0 | | 0.89 | 0.74 |
| *Mentzelia nitens* | 0.00 | 0.00 |  | | 0.20 | <0.01 |  | | 0.63 | 0.66 |
| *Salvia columbariae* | 0.00 | 0.00 |  | | 0.00 | 0.00 |  | | 1.00 | 0.60 |
| *Eriastrum densifolium* | 0.13 | <0.01 |  | | 0.00 | 0.00 |  | | 0.88 | 0.61 |
| *Populus* | 0.00 | 0.00 |  | | 0.00 | 0.00 |  | | 1.00 | 1.00 |
| *Yucca brevifolia* | 0.00 | 0.00 | 0.01 | | 0.20 | <0.01 | 0.11 | | 0.70 | 0.01 |
| *Leptosyne* | 0.00 | 0.00 |  | | 0.00 | 0.00 |  | | 1.00 | 1.00 |
| *Ephedra* | 0.13 | <0.01 | 0.11 | | 0.00 | 0.00 | <0.01 | | 0.86 | 0.16 |
| *Chenopodium* | 0.00 | 0.00 |  | | 0.00 | 0.00 |  | | 1.00 | 1.00 |
| *Camissonia campestris* | 0.00 | 0.00 |  | | 0.20 | <0.01 |  | | 0.65 | 0.60 |
| *Stephanomeria* | 0.13 | <0.01 |  | | 0.00 | 0.00 |  | | 0.85 | 0.80 |
| *Descurainia pinnata* | 0.00 | 0.00 |  | | 0.00 | 0.00 |  | | 1.00 | 0.67 |
| *Cuppressaceae* | 0.00 | 0.00 |  | | 0.00 | 0.00 |  | | 1.00 | 1.00 |
| *Cirsium arvense* | 0.13 | <0.01 |  | | 0.00 | 0.00 |  | | 0.86 | 0.58 |
| *Erodium cicutarium* | 0.00 | 0.00 |  | | 0.00 | 0.00 |  | | 1.00 | 0.25 |
| *Claytonia sp* | 0.00 | 0.00 |  | | 0.00 | 0.00 |  | | 1.00 | 1.00 |
| *Poaceae* | 0.00 | 0.00 |  | | 0.00 | 0.00 |  | | 1.00 | 0.26 |
| *Lupinus* | 0.00 | 0.00 |  | | 0.00 | 0.00 |  | | 1.00 | 0.34 |
| *Ceanothus cordulatus* | 0.13 | <0.01 |  | | 0.00 | 0.00 |  | | 0.82 | 0.89 |
| *Phragmites australis* | 0.00 | 0.00 |  | | 0.00 | 0.00 |  | | 1.00 | 0.73 |
| *Camissonia kernensis* | 0.00 | 0.00 |  | | 0.00 | 0.00 |  | | 1.00 | 0.60 |
| *Thysanocarpus* | 0.00 | 0.00 |  | | 0.00 | 0.00 |  | | 1.00 | 0.89 |
| *Scrophularia desertorum* | 0.13 | <0.01 |  | | 0.00 | 0.00 |  | | 0.86 | 0.85 |
| *Bromus* | 0.00 | 0.00 |  | | 0.00 | 0.00 |  | | 1.00 | 0.60 |

**Table S3:** Frequency of occurrence (FOO) and relative read abundance (RRA) of 33 unique plant taxa identified in the diets of *N. bryanti* and *N. lepida* in spring and summer 2016 combined. *P-*values are corrected for multiple comparisons. We confirmed the presence of *E. nauseosa* voucher sequences in some samples and therefore include these within the Asteraceae family. Where applicable, the percent abundance of woody plants in each habitat is reported.

| **Taxa Identified** | ***N. bryanti* (n = 19)** | | |  | ***N. lepida* (n = 16)** | | |  | ***P-value*** | |
| --- | --- | --- | --- | --- | --- | --- | --- | --- | --- | --- |
|  | FOO | RRA | %hill | | FOO | RRA | %flats | | FOO | RRA |
| *Prunus fasciculata* | 0.21 | 0.04 | 0.04 | | 1.00 | 0.79 | 0.10 | | **0.01** | **0.01** |
| *Frangula californica* | 0.89 | 0.41 | 0.13 | | 0.06 | <0.01 | 0.01 | | **0.01** | **0.01** |
| *Phacelia tanacetefolia* | 0.89 | 0.11 |  | | 0.82 | 0.14 |  | | 0.65 | 0.34 |
| *Pinus* | 0.84 | 0.19 | 0.05 | | 0.12 | <0.01 | <0.01 | | **0.01** | **0.01** |
| *Eriogonum umbellatum* | 0.68 | 0.08 |  | | 0.24 | 0.03 |  | | **0.01** | 0.16 |
| *Ribes amarum* | 0.32 | 0.04 | 0.03 | | 0.00 | 0.00 | 0 | | 0.06 | **0.01** |
| *Acmispon americanus* | 0.32 | 0.03 |  | | 0.00 | 0.00 |  | | 0.08 | **0.03** |
| *Asteraceae* | 0.37 | 0.02 |  | | 0.12 | <0.01 |  | | 0.14 | 0.17 |
| *Ericameria nauseosa_voucher_* | 0.21 | <0.01 | 0.33 | | 0.00 | 0.00 | 0.60 | | NA | NA |
| *Euphorbia maculata* | 0.00 | 0.00 |  | | 0.24 | 0.02 |  | | 0.10 | 0.06 |
| *Cercocarpus betuloides* | 0.16 | 0.01 | <0.01 | | 0.00 | 0.00 | 0 | | 0.23 | 0.29 |
| *Mentzelia nitens* | 0.11 | <0.01 |  | | 0.12 | <0.01 |  | | 0.88 | 0.71 |
| *Salvia columbariae* | 0.21 | <0.01 |  | | 0.00 | 0.00 |  | | 0.20 | 0.10 |
| *Eriastrum densifolium* | 0.11 | <0.01 |  | | 0.00 | 0.00 |  | | 0.44 | 0.15 |
| *Populus* | 0.05 | <0.01 |  | | 0.00 | 0.00 |  | | 0.75 | 0.74 |
| *Yucca brevifolia* | 0.00 | 0.00 | 0.01 | | 0.12 | <0.01 | 0.11 | | 0.34 | **0.01** |
| *Leptosyne* | 0.11 | <0.01 |  | | 0.00 | 0.00 |  | | 0.47 | 0.25 |
| *Ephedra* | 0.11 | <0.01 | 0.11 | | 0.00 | 0.00 | <0.01 | | 0.50 | **0.01** |
| *Chenopodium* | 0.00 | 0.00 |  | | 0.06 | <0.01 |  | | 0.72 | 0.41 |
| *Camissonia campestris* | 0.00 | 0.00 |  | | 0.12 | <0.01 |  | | 0.34 | 0.09 |
| *Stephanomeria* | 0.11 | <0.01 |  | | 0.00 | 0.00 |  | | 0.45 | 0.07 |
| *Descurainia pinnata* | 0.00 | 0.00 |  | | 0.06 | <0.01 |  | | 0.75 | **0.04** |
| *Cuppressaceae* | 0.05 | <0.01 |  | | 0.00 | 0.00 |  | | 0.76 | 0.80 |
| *Cirsium arvense* | 0.05 | <0.01 |  | | 0.00 | 0.00 |  | | 0.72 | 0.17 |
| *Erodium cicutarium* | 0.00 | 0.00 |  | | 0.06 | <0.01 |  | | 0.75 | **0.02** |
| *Claytonia sp* | 0.11 | <0.01 |  | | 0.00 | 0.00 |  | | 0.45 | **0.01** |
| *Poaceae* | 0.00 | 0.00 |  | | 0.06 | <0.01 |  | | 0.71 | 0.27 |
| *Lupinus* | 0.05 | <0.01 |  | | 0.00 | 0.00 |  | | 0.80 | **0.02** |
| *Ceanothus cordulatus* | 0.05 | <0.01 |  | | 0.00 | 0.00 |  | | 0.80 | 0.83 |
| *Phragmites australis* | 0.00 | 0.00 |  | | 0.06 | <0.01 |  | | 0.71 | 0.25 |
| *Camissonia kernensis* | 0.00 | 0.00 |  | | 0.06 | <0.01 |  | | 0.72 | **0.02** |
| *Thysanocarpus* | 0.05 | <0.01 |  | | 0.00 | 0.00 |  | | 0.75 | 0.45 |
| *Scrophularia desertorum* | 0.05 | <0.01 |  | | 0.00 | 0.00 |  | | 0.72 | 0.16 |
| *Bromus* | 0.00 | 0.00 |  | | 0.06 | <0.01 |  | | 0.71 | 0.29 |

**
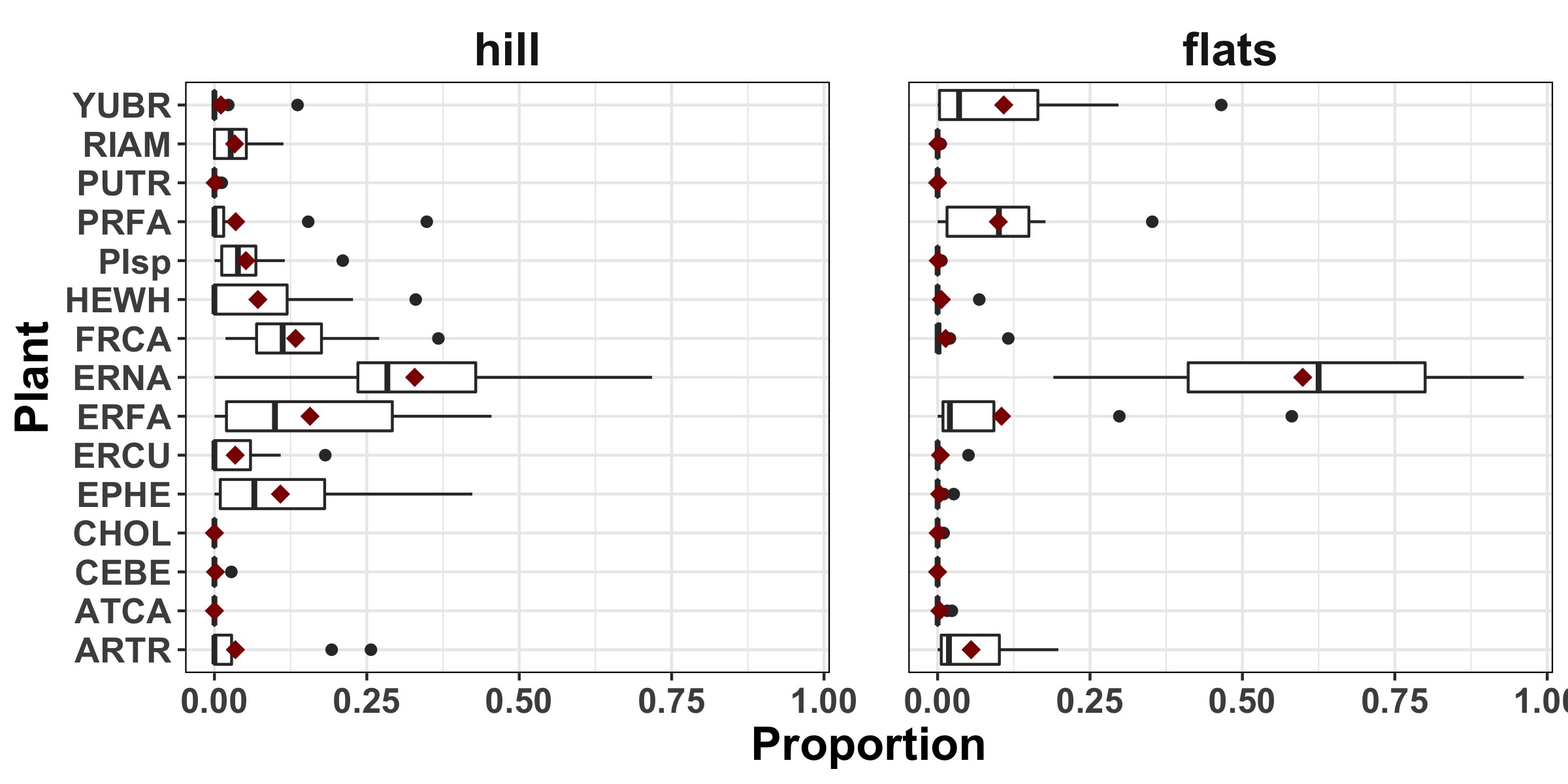
Figure S1:** Relative proportion of shrubs and trees present at the study site estimated from counts within plots in both hill and flats. Black bars are median values; dark red diamonds are the mean of each plant in that habitat. Four-letter codes: YUBR – *Yucca brevifolia*; RIAM – *Ribes amarum*; PUTR – *Purshia tridentata*; PRFA – *Prunus fasciculata*; PIsp – *Pinus* sp.; HEWH – *Hesperoyucca whipplei*; FRCA – *Frangula californica*; ERNA – *Ericameria nauseosa*; ERFA – *Eriogonum fasciculatum*; ERCU *– Ericameria cuneata*; EPHE – *Ephedra* sp.; CHOL – *Cholla* sp.; CEBE – *Cercocarpus betuloides;* ATCA – *Atriplex canescens* ; ARTR – *Artemisia tridentata*.

**Table S4**: Average values of relative proportion of the 15 common shrubs and trees in the hill and flats habitat shown in Figure S1.

| **Plant** | **hill** | **flats** |
| --- | --- | --- |
| YUBR | 0.011 | 0.108 |
| RIAM | 0.033 | 0 |
| PUTR | 0.001 | 0 |
| PRFA | 0.035 | 0.1 |
| PI*sp.* | 0.052 | 0.001 |
| HEWH | 0.071 | 0.006 |
| FRCA | 0.133 | 0.013 |
| ERNA | 0.329 | 0.599 |
| ERFA | 0.157 | 0.105 |
| ERCU | 0.034 | 0.005 |
| EPHE | 0.108 | 0.003 |
| CHOL | 0 | 0.001 |
| CEBE | 0.002 | 0 |
| ATCA | 0 | 0.004 |
| ARTR | 0.034 | 0.055 |

**Table S5**: Bayesian posterior probabilities (medians and 95% credible intervals) of relative consumption of plants identified in woodrat diets from 2016. Raw relative read counts from trn*L* sequences were used to estimate population level consumption of plants using *bayespref*. The 5 listed plants here comprise > 90% of the total reads of 33 total plants identified. The “Other” category contains the sum of all reads of the remaining 28 plants in dataset.

|  |  | |  |  | |  |  | |  |
| --- | --- | --- | --- | --- | --- | --- | --- | --- | --- |
|  | **Spring** | |  | **Summer** | |  | **Combined** | |  |
| **Plant** | ***N. bryanti* (n = 11)** | ***N. lepida***  **(n = 11)** | | ***N. bryanti***  **(n = 8)** | ***N. lepida***  **(n = 5)** | | ***N. bryanti***  **(n = 19)** | ***N. lepida***  **(n = 16)** | |
| *Prunus fasciculata* | 0.05 (0.03, 0.09) | 0.54 (0.45, 0.61) | | 0.06 (0.03, 0.10) | 0.65 (0.56, 0.71) | | 0.05 (0.03, 0.08) | 0.58 (0.51, 0.64) | |
| *Frangula californica* | 0.22 (0.14, 0.30) | 0.03 (0.01, 0.05) | | 0.36 (0.26, 0.45) | 0.01 (0.00, 0.02) | | 0.27 (0.20, 0.34) | 0.02 (0.01, 0.03) | |
| *Phacelia tanacetefolia* | 0.15 (0.09, 0.23) | 0.19 (0.11, 0.26) | | 0.15 (0.09, 0.23) | 0.13 (0.09, 0.19) | | 0.16 (0.11, 0.21) | 0.17 (0.13, 0.22) | |
| *Pinus sp.* | 0.17 (0.10, 0.26) | 0.04 (0.02, 0.07) | | 0.19 (0.12, 0.27) | 0.03 (0.01, 0.07) | | 0.19 (0.13, 0.25) | 0.04 (0.02, 0.06) | |
| *Eriogonum umbellatum* | 0.12 (0.07, 0.19) | 0.04 (0.02, 0.07) | | 0.08 (0.04, 0.14) | 0.04 (0.02, 0.08) | | 0.11 (0.07, 0.15) | 0.04 (0.02, 0.07) | |
| *Other* | 0.27 (0.18, 0.37) | 0.15 (0.10, 0.22) | | 0.15 (0.09, 0.22) | 0.13 (0.08, 0.19) | | 0.22 (0.16, 0.28) | 0.14 (0.11, 0.19) | |
